# Biomimetic Surfaces Coated with Covalently Immobilized Collagen Type I: An X-Ray Photoelectron Spectroscopy, Atomic Force Microscopy, Micro-CT and Histomorphometrical Study in Rabbits

**DOI:** 10.3390/ijms20030724

**Published:** 2019-02-08

**Authors:** Antonio Scarano, Felice Lorusso, Tiziana Orsini, Marco Morra, Giorgio Iviglia, Luca Valbonetti

**Affiliations:** 1 Department of Medical, Oral and Biotechnological Sciences, Center for Research on Aging and Translational Medicine (CeSI-MeT), University of Chieti-Pescara, 66100 Chieti, Italy; drlorussofelice@gmail.com; 2 Research staff at Zirconia Implant Research Group (Z.I.R.G), International Academy of Ceramic Implantology, USA; 3 CNR—National Research Council, Institute of Cell Biology and Neurobiology (IBCN), 00015 Roma, Italy; tiziana.orsini@cnr.it; 4 Nobil Bio Ricerche Srl, 14037 Portacomaro (AT), Italy; mmorra@nobilbio.it (M.M.); giviglia@nobilbio.it (G.I.); 5 Department of Applied Science and Technology, Institute of Materials Physics and Engineering, Politecnico di Torino, 10121 Torino, Italy; 6 Faculty of Bioscience and Agro-Food and Environmental Technology, University of Teramo, 64100 Teramo, Italy; lvalbonetti@unite.it

**Keywords:** collagen type I, implant surfaces, bone implant contact, hydrophilic surface, micro CT

## Abstract

Background: The process of osseointegration of dental implants is characterized by healing phenomena at the level of the interface between the surface and the bone. Implant surface modification has been introduced in order to increase the level of osseointegration. The purpose of this study is to evaluate the influence of biofunctional coatings for dental implants and the bone healing response in a rabbit model. The implant surface coated with collagen type I was analyzed through X-ray Photoelectron Spectroscopy (XPS), Atomic Force Microscopy (AFM), micro-CT and histologically. Methods: The sandblasted and double acid etched surface coated with collagen type I, and uncoated sandblasted and double acid etched surface were evaluated by X-ray Photoelectron Spectroscopy (XPS) and Atomic Force Microscopy (AFM) analysis in order evaluate the different morphology. In vivo, a total of 36 implants were positioned in rabbit articular femoral knee-joint, 18 fixtures for each surface. Micro-CT scans, histological and histomorphometrical analysis were conducted at 15, 30 and 60 days. Results: A histological statistical differences were evident at 15, 30 and 60 days (*p* < 0.001). Both implant surfaces showed a close interaction with newly formed bone. Mature bone appeared in close contact with the surface of the fixture. The AFM outcome showed a similar roughness for both surfaces. Conclusion: However, the final results showed that a coating of collagen type I on the implant surface represents a promising procedure able to improve osseointegration, especially in regions with a low bone quality.

## 1. Introduction

Dental implants are successfully applied for partially or totally edentulous patients with missing single or multiple teeth [1]. The osteointegration rate, bone quality and bone in contact with the dental implant influence long-term success of oral implant rehabilitation [2,3]. One of the prerequisites for success of an implant in oral rehabilitation is bone implant contact, bone density and quality around titanium. Different implant surface treatments have been proposed by different authors to increase bone implant contact and bone density. Micro- and macro-porous structures of titanium surfaces have been evaluated to promote an enhanced peri-implant bone apposition during the early stages of bone formation. Numerous animal studies have shown how the microstructure increases removal torque [4] and macrostructure surface increases angiogenesis [5]. For roughening titanium implant surfaces different methods are used and classified by subtraction and addition into mechanical, chemical and physical methods. Subtractive techniques can be used such as sand-blasting with abrasives on TiO_2_, AlO_2_, hydroxyapatite (HA), Laser, electrochemical deposition, acid-etching or dual acid-etching, or combinations of such treatments or organic biomaterials. The biomaterials used for implant surface modification have been HA, CaP or micro/nano coating. All of these implant surface treatments increased cell proliferation and growth, improved wettability and accelerated the osseointegration process [6,7]. In vitro [8] and in vivo [9] studies have demonstrated that micro or macro implant surfaces play a pivotal role, enhancing osteoblastic behavior and responses, such as increased cell attachment, proliferation in bone implant contact and bone quality and density. New surfaces have been proposed by different authors; for example a post-machining surface after treatment with sandblasting and acid-etching or dual acid-etching was studied through a variety of techniques or the incorporation of bioactive ceramic, HA and calcium phosphate (CaP), crystals polymer coatings [10] or polyetheretherketone (PEEK) coating [11]. These coatings improved and increased the surface area of the implants and increased dental implant stability and bone anchoring/biomechanics [12].

Despite the excellent results obtained with the new implant surfaces in improving osseointegration, the surface modification of Ti coatings is still an interesting viable. Recently, thermal treatment has been proposed for improving the bioactivity of implant surfaces with increase the bone implant contact [13]. 

A new strategy to improve osseointegration is that of the immobilization on Ti of the main components of the extracellular matrix, peptides or enzymes such as type I collagen for promoting the adhesion of osteoblasts [14]. Collagen type I has been added onto implant surfaces when it is present in the extracellular matrix of bone showing osteoconductivity, biocompatibility activity and it performs the function of support by giving structural support to resident cells and ductility to the bone [15]. Collagen type I can be one of the prime candidate materials for realizing tissue-engineered grafts and is one of the proteins that work critical roles in bone mineralization [16], bone healing [17], improving blood compatibility and osteoblastic adhesion, differentiation, and extracellular-matrix secretion [18]. The present study was based on the hypothesis that collagen type I improves bone implant contact (BIC) and osseointegration.

The purpose of this study was to characterize two different surfaces by X-ray Photoelectron Spectroscopy (XPS) and Atomic Force Microscopy (AFM) and compare, in a rabbit model, the BIC, bone area inner threads (BAIT) and bone area outer threads (BAOT) by histologic analysis and micro CT between collagen coated and uncoated implant surfaces using a split implant design. The null hypothesis stated that there are no statistically significant differences in BIC, BAIT, BAOT and osseointegration cell responses between collagen coated and uncoated implants.

## 2. Results

### 2.1. Surface Analysis by X-Ray Photoelectron Spectroscopy (XPS)

#### 2.1.1. Surface (Uncoated Implant)

**Figure 1:**
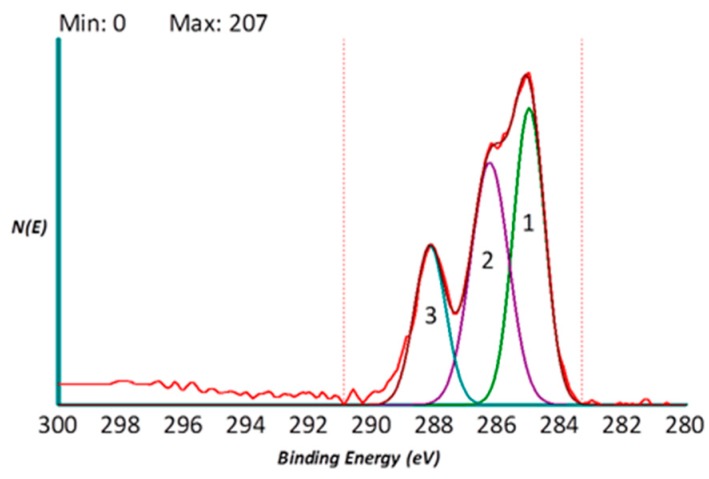
High resolution C1s peak obtained by XPS of ColTi sample. The figure shows the experimental curve (red) and the sum curve (brown) resulting from fitting with 1 (C–C, C–H) (green), 2 (C–N, C–O) (violet) and 3 (C=O, N–C=O functionalities) (azure).

The control group of samples were only sandblasted and acid etched. The XPS-detected surface composition of uncoated and collagen coated samples is shown in Table 1. Briefly, the surface of titanium is covered by a thin (about 4 nm thick) oxide layer, so that the maximum theoretical concentration of Ti on pure titanium is 33%, the rest being oxygen (the most stable oxide is TiO2). Surface contamination by adsorption of ubiquitous hydrocarbons from the atmosphere introduces a surface overlayer of carbon, readily captured by surface-sensitive techniques such as XPS, decreasing the concentration of Ti below the theoretical value (Figure 1). 

#### 2.1.2. Surface (Coated Collagen Type I Implant)

Observing the collagen coated sample, a completely different surface stoichiometry is detected. The signal from titanium almost disappears, while O/Ti and C/Ti ratio increase and nitrogen rises up to over 14%. This is a clear indication that the coating process introduces on the surface an organic molecule featuring multiple carbon-oxygen and carbon-nitrogen functionalities. Further clues are supplied by the high-resolution C1 peak of the collagen coated sample shown in Figure 1, the experimental curve (red) is highly asymmetric and made up of different components.

Figure 1 also shows the results of peak deconvolution, according to literature procedures and general XPS approach to extract information on the carbon chemical environment. The peak is nicely fitted by three components located at 285.00 eV (component 1, C–C, C–H), 286.3o eV (component 2, C–N, C–O), and 288.10 eV (component 3, C=O, N-C=O functionalities). So, the combination of surface stoichiometry (Table 1) and curve fitting data indicates that the surface of the coated implant contains an organic layer that, beside C–O and C–N bonds, is rich in amide-type functionalities (component 3 at 288.10 eV) and confirms that collagen molecules coat the titanium surface chemistry. (Table 1) Incidentally, the small signal from titanium detected in the survey spectrum (Table 1), suggests that the thickness of the coating is lower than the XPS-sampling depth (8 nm) (Figure 1).

### 2.2. Atomic Force Microscopy (AFM)

**Figure 2:**
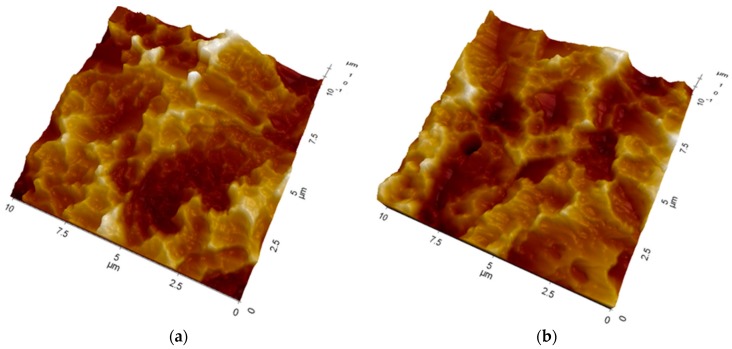
10 × 10 micrometer images obtained by non-contact AFM analysis of control (**a**) and collagen coated (**b**) Ti disks subjected to the same treatment as the tested implants.

In both cases the topography of the disks shows the typical microroughness imparted by double acid etching (the field of view is too small to also detect the longer-range roughness due to sandblasting). No evidence of the organic layer detected by XPS is observed (Figure 2a). That is, even if the vertical resolution of AFM is sub-nanometer, the microrough surface topography does not allow image adsorbed collagen macromolecules (Figure 2b), which are instead plainly detected on flat surfaces [19]. For 10 × 10 micrometer areas, the Sdr roughness parameter (that indicates the percentage increase of actual surface area with respect to the geometrical one) is 88 ± 7 for control sample and 85 ± 3 for the collagen coated one, without significant difference. On one hand, this result confirms that the process does not involve the deposition of a thick coating, but of a conformal, nanometer-thin surface layer. On the other hand, and from the point of view of the present study, this observation suggests that whatever difference is detected in implantation tests it is not dictated by differences in surface topography, rather, it genuinely reflects the contribution of the different surface chemistries to peri-implant new bone formation.

### 2.3. Micro-CT Evaluation

Micrographs were evaluated for bone BIC, BAIT, BAOT and gaps between bone and implant. Radiographs showed new bone in both implants in intimate contact with the implant surface, no gaps were detected at 15 days. At 30 days the BIC and BAIT were more present in the coated implant with collagen I. No signs of bone resorption inflammation and/or osteolysis were observed in either surfaces.

### 2.4. Histological Evaluation

Under light microscopy, all 36 implants were surrounded by bone and showed good osseointegration. The lower threads of the implants appeared to be in close contact with newly formed bone or with marrow spaces, while along the upper threads there was contact with the cortical bone. No evidence of soft tissue was present between bone and implant surfaces in all the specimens of either experimental groups.

#### 2.4.1. Fifteen Days

##### Uncoated Implant Surface

**Figure 3:**
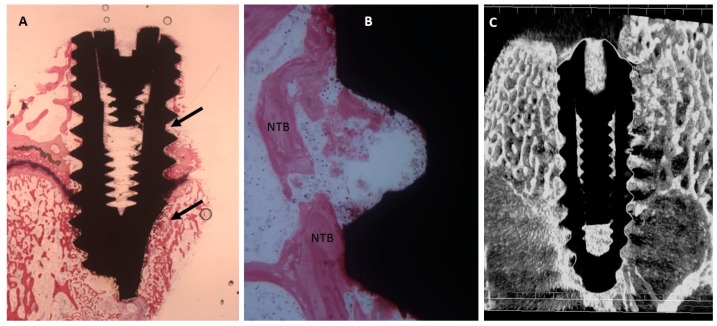
(**A**) Dental implants surrounded by the trabeculae of newly-formed bone. Toluidine blue and acid fuchsin staining 2×. (**B**) Higher magnification. A few bone trabeculae were present in the implant concavities (NTB). Toluidine blue and acid fuchsin 50×. (**C**) Micro-CT scans realized along the transection planes of the dental implant. Newly-formed bone was present.

The slide observed at low magnification showed a few trabeculae bone near or in contact with the implant surface (Figure 3). Also, osteoblasts were present near and in contact with the implant. Few inflammatory cells were observed at the interface implant bone. 

The mean BIC percentage was 22.42 ± 4.5%, bone area inner threads (BAIT) was 23 ± 0.8% and bone area outer threads (BAOT) was 19 ± 0.8%.

##### Collagen-Coated Surface

**Figure 4:**
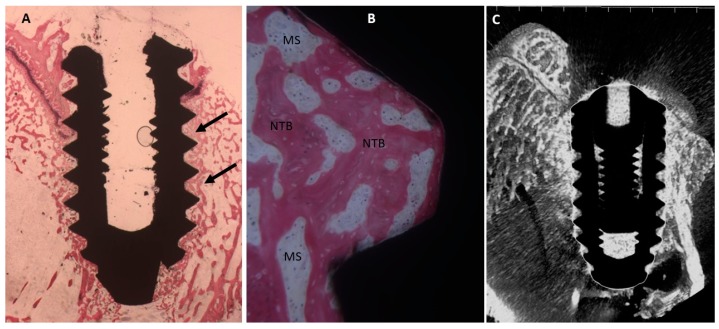
(**A**) New bone was present around the implants and in the implant concavities (arrows). Toluidine blue and acid fuchsin staining 2×. (**B**) At higher magnification, more trabeculae bone was present in the implant concavities (NTB) with a small medullary space (MS). Toluidine blue and acid fuchsin staining 50×. (**C**) Micro-CT scans along transection planes of dental implant. A newly-formed bone was present.

It was possible to observe a trabecular bone in contact with the fixture surface (Figure 4). This trabecular is present in the concavity of the implant. At high magnification it is possible to observe many osteoblasts in direct contact with the titanium. The mean BIC percentage was 27.5 ± 3.1%, bone area inner threads (BAIT) was 31 ± 0.8% and bone area outer threads (BAOT) was 21.8 ± 1%.

#### 2.4.2. Thirty Days

##### Uncoated Implant Surface 

**Figure 5:**
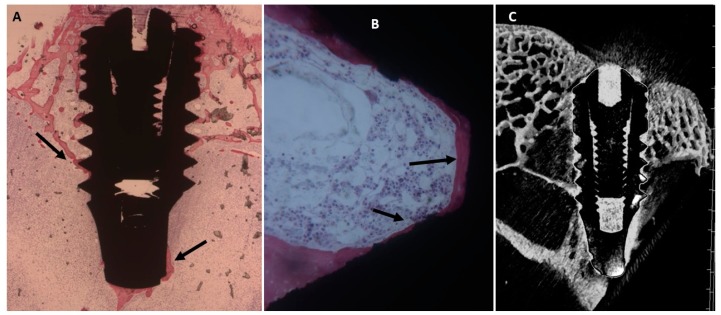
(**A**) The Histological evaluation showed organization and mineralization of the bone tissue especially near the dental implant (arrows). Toluidine blue and acid fuchsin staining 2×. (**B**) At higher magnification, a thin trabecula without osteoblastic activity was present in the implant concavities (arrows). Toluidine blue and acid fuchsin staining 50×. (**C**) Micro-CT scans along transection planes of the dental implant. Bone was present in contact with the dental implant.

The slide showed mature bone in contact with the implant, in only a few areas it is possible to observe osteoblasts (Figure 5). No pathological infiltrate of inflammatory cells was observed. 

The mean BIC percentage was 51.2 ± 3.9%, bone area inner threads (BAIT) was 28 ± 0.8% and bone area outer threads (BAOT) was 36 ± 0.8%.

##### Collagen-Coated Surface

**Figure 6:**
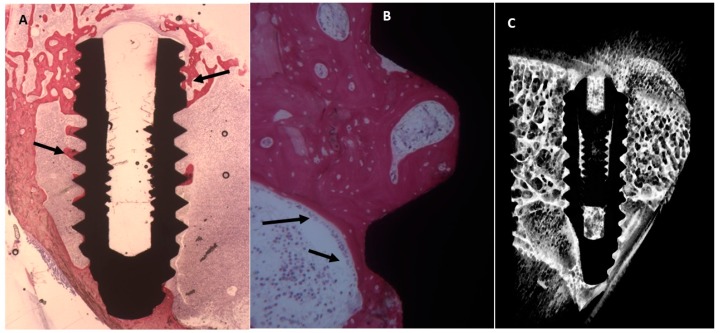
(**A**) Bone with complete organization and mineralization especially near the dental implant (arrows). Toluidine blue and acid fuchsin staining 2×. (**B**) At higher magnification, many osteoblasts (arrows) and osteoid matric were present in the fixture concavities. Toluidine blue and acid fuchsin staining 50×. (**C**) Micro-CT images along transection planes of the dental implant. Mature bone was present in contact with the dental implant.

Mature bone was found on the surface of titanium with cylindrical structures that are haversian system. No gaps were observed between implant and bone. A little osteoblast activity was present (Figure 6).

The mean BIC percentage was 55.3 ± 3.2%, bone area inner threads (BAIT) was 39 ± 2.2% and bone area outer threads (BAOT) was 38 ± 2.2%.

#### 2.4.3. Sixty Days

##### Uncoated Implant Surface 

**Figure 7:**
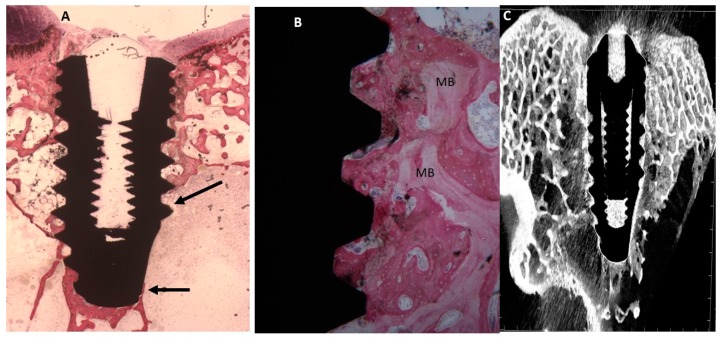
**(A**) A thin layer of mature bone was present around the fixture. Toluidine blue and acid fuchsin staining 2×. (**B**) At higher magnification, a remodeling mature bone (MB) was evident around the implant surface (Arrows). Toluidine blue and acid fuchsin staining 50×. (**C**) Micro-CT scans along transection planes of the dental implant. Trabecular bone was present in contact with the dental implant.

At lower magnification, evidence of a mature bone was observed in contact with the implant surface. A few osteoblast activities were present (Figure 7). The mean BIC percentage was 56.32 ± 3.2%, bone area inner threads (BAIT) was 35 ± 2.3% and bone area outer threads (BAOT) was 36 ± 2.3%.

##### Collagen-Coated Surface

**Figure 8:**
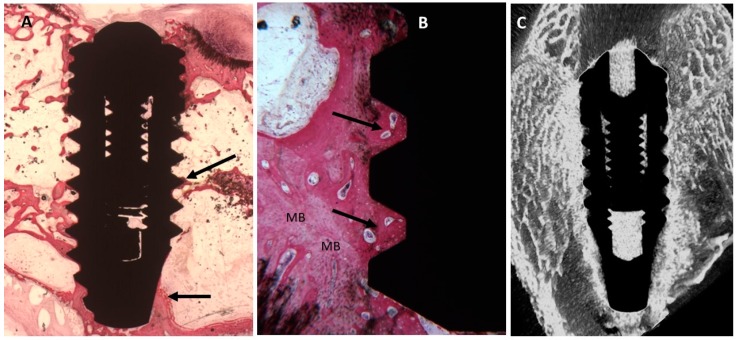
(**A**) Trabecular bone was more abundant around the dental implant (arrows). Toluidine blue and acid fuchsin staining 2×. (**B**) At higher magnification, mature bone (MB) was present around the implant surface and the concavities were completely filled by new mature bone (Arrows). Toluidine blue and acid fuchsin staining 50×. (**C**) Micro-CT scans along transection planes of the dental implant. Trabecular bone was present in contact with the dental implant and was also more than that in the control implant.

Low magnification showed mature bone tissues along the periphery of the implant and in the thread of the implant. A few osteoblasts were also found around the titanium. No gaps nor inflammatory nor multinucleate cells were detected near the titanium surface (Figure 8). The mean BIC percentage was 63.6 ± 2.9%, bone area inner threads (BAIT) was 42 ± 2.3% and bone area outer threads (BAOT) was 44 ± 2.3%.

### 2.5. Statistical Evaluation

The means and SD values of BIC, BAIT and BAOT ratios are shown in Table 2. Significant differences were found in the BIC, BAIT and BAOT ratios at 15, 30 and 60 days (*p* < 0.05).

**Figure 9:**
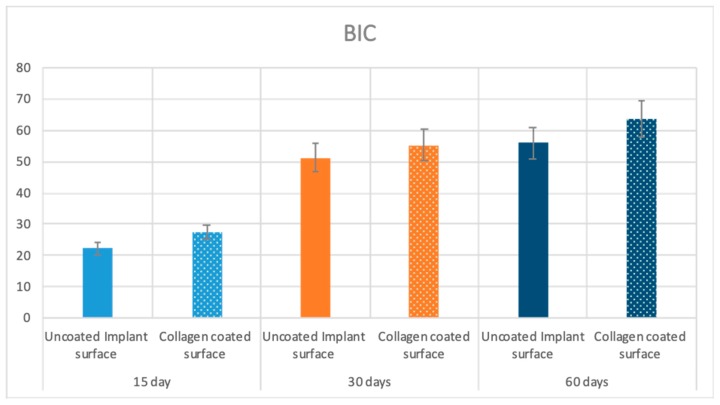
Means and SD of the measured BIC ratios at 15, 30 and 60 days. Y axis is bone implant contact expressed in %.

**Figure 10:**
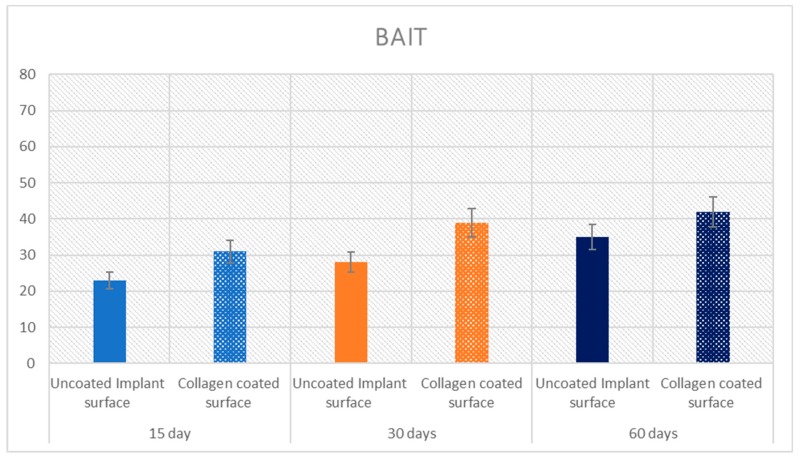
Means and SD of the measured BAIT ratios at 15, 30 and 60 days. Y axis is BAIT expressed in %.

**Figure 11:**
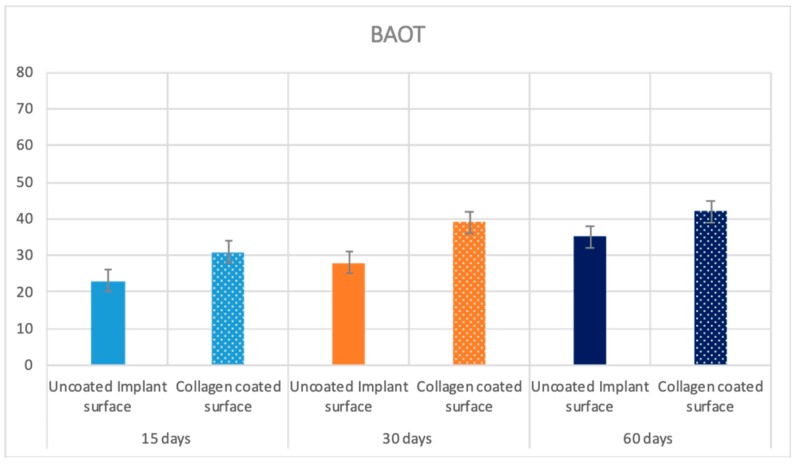
Means and SD of the measured BAOT ratios at 15, 30 and 60 days. Y axis is BAOT implant contact expressed in %.

The Pearson correlation test found significant correlation between the bone implant contact (BIC), bone area inner threads (BAIT) and bone area outside threads (BAOT) ratios for either 15, 30 and 60 days (*p* < 0.05). (Table 2 and Table 3; Figure 9, Figure 10 and Figure 11).

## 3. Discussion

The aim of this study was to investigate the effects of collagen type I on bone healing and bone formation in a rabbit model when used as coating on titanium implants. The investigators hypothesized that dental implant coated with collagen type I may not increase the amount of bone in contact with the implant surface.

Even if many in vivo and vitro studies have investigated collagen type I on titanium, there is a lack of information on how such a coated surface influences BIC, BAIT and BAOT. The outcomes of the XPS evaluation show that titanium disk surfaces evaluated in the present study are in good agreement with the literature on titanium surfaces in general and, in particular, on surfaces of titanium dental implants [20]. A large amount of literature exists on this subject, the value of about 18% Ti detected in this case is indicative of a comparatively clean surface [21,22].

In the coated disks the XPS shows the presence of a proteinaceous layer on the implant surface and it obviously confirms that collagen molecules coat the titanium surface chemistry. In a few areas, a small signal from titanium in the survey spectrum is detected (Table 1), suggesting that the thickness of the coating is lower than the XPS-sampling depth (8 nm). Again, this is consistent with the nature of the process, which is based on interfacial adsorption of collagen molecules rather than the application of a thick coating. Titanium surfaces coated with different substances as in dental implants have attracted increasing attention.

In this study sandblasted and acid-etched implant screws, uncoated or coated with collagen type I were tested in a knee implantation model in rabbit. The outcomes of the present research showed a statistical difference in BIC, BAIT BAOT between titanium uncoated or coated with collagen type I. The results of the present study support the rejection of the null hypothesis. The collagen coating probably influences cell migration, attachment, proliferation, and differentiation on the titanium implants, but no bone formation distant from the implant surface. These results are not influenced by roughness, in fact the AFM outcome showed a similar roughness surface for both implants. These findings were confirmed by Micro-CT. Type I collagen on the surface of titanium implants improved early osteogenesis [23,24,25,26]. It was seen that collagen type I improved osseointegration and postcondition of titanium coated implants. Similar results were obtained in a rabbit model with a femur condyle defect when the implants were coated in collagen type I [17]. It follows that coating biomolecules positively influence mesenchymal cell proliferation, attachment, and differentiation [24,27]. The positive influence of collagen coating was confirmed in a mini pig model. In the experimental model, the authors showed increased bone implant contact and bone density [26]. In this study a rabbit model was chosen, where the knee served well for comparison of osseointegration between different implant groups. Collagen type I improved bone inside the screw threads and in contact with the surface when the implants were placed in the rabbit femur trabecular bone [28]. Schliephake et al. studied collagen-coated implants and demonstrated an increase in the percentage of BIC in a dog model [29]. Also, the study of Bae (2018) [30] in a rat tibia model showed an enhancement in the osseointegration and bone healing with collagen type I crosslinked by gamma irradiation. In this research we have not investigated what happens to the collagen during the phases of osseointegration; it is probably resorbed during the phases of bone healing. The collagen type I it could offer greater resistance to pre-implantitis and were used for treating the hard tissue loss associated with peri-implantitis around SLA implants [31].

We used the rabbit model because it is a convenient model for skeletal research studies and has been extensively used to test biomaterials [32] and osteoconductive/osteoinductive reactions in implants [33]. Besides, this animal model provides an excellent cost-effective animal model, their maintenance and housing are simple, and they recover very well postoperatively. In addition the bone composition and morphology between rabbit and human are very similar [34], and there is a sufficient correlation with maxilla bone [35]. This model was also chosen because the phenomenon of osseointegration was discovered in the rabbit [36] and for this advantage they are often used in skeletal research or for screening implant materials.

In this study, we chose a rabbit femur model to evaluate the potential ability of coating surfaces to enhance bone formation when coated by collagen in the presence of large medullary spaces.

## 4. Materials and Methods

### 4.1. Surface Preparation by the Manufacturer

The surfaces evaluated in this investigation were prepared by the manufactures. Tests were performed on grade 4 control and collagen coated dental implants. Both of them were sandblasted and acid etched. The uncoated surface shows the typical topography of sandblasted-acid etched surfaces, featuring a long-range roughness due to the plastic deformation of the surface induced by blasting, and a short-range roughness due to acid etching. The Sa roughness value, as evaluated by Stereo Scanning Microscopy analysis of 110 × 80 micrometers areas, is around 1.15 micrometers. 

Collagen coated samples were further subjected to a proprietary treatment, based on the interfacial adsorption and linking of collagen molecules to titanium, yielding a nanometer-thin, permanent molecular layer on the implant surface. As discussed in the relevant section, no detectable modification of surface topography and roughness, at the micrometer level, is induced by the collagen nanolayer. Purified bovine collagen Type I, dry, obtained from calf hides through pepsin extraction, supplied by Symatese (Chaponost, France) is used in the process. The product complies with the European requirements: Regulation (UE) N° 722/2012 of 8 August 2012 with respect to active implantable medical devices and medical devices manufactured utilizing tissues of animal origin; and it has obtained a certificate of suitability with respect to Monograph N° 1483 of European Pharmacopieia: “Products with risk of transmitting agents of animal spongiform encephalopathies”. Dry collagen is dissolved in acidic (acetic acid) solution on coating process.

### 4.2. Surface Analysis by X-Ray Photoelectron Spectroscopy (XPS)

The roughness of the coated and uncoated implants was analyzed using atomic force microscopy. The disks used in the present study were sandblasted and double acid etched.

Ten titanium sandblasted and double acid etched disks and ten titanium sandblasted and double acid etched disks and coated with collagen type I were used for the evaluation of the surface composition of uncoated and collagen coated disks by XPS analysis. All samples were titanium grade 4 with 5 mm diameter subjected to the same treatments as implants. 

XPS analysis was performed by a Perkin Elmer PHI 5600 ESCA system (PerkinElmer Inc., Waltham, MA, USA). The instrument is provided with a monochromatized Al anode operating at 10 kV and 200 W. The diameter of the analyzed spot is about 500 micrometers, the analyzed depth about 8 nanometers. The base pressure was maintained at 10 Pa. The angle between the electron analyzer and the sample surface was 45°. Analysis was performed by acquiring wide-range survey spectra (0–1000 eV binding energy), using a pass energy of 117.4 eV, resolution of 1 eV/Step and acquisition time of 50 ms/Step. After acquisition of the survey spectra, high resolution C1s peaks were acquired using a pass energy of 11.75 eV, resolution of 0.100 eV/Step and acquisition time of 50 ms/Step. Deconvolution of C1s peak was performed using the instrument’s software, after correction of the peak position with reference to the internal standard C–C component of the C1s peak positioned at 285.0 eV.

### 4.3. Atomic Force Microscopy (AFM)

Ten SLA titanium disks and ten titanium disks coated with collagen type I were studied for the analysis of the surface topography by AFM. All of the disks were titanium grade 4 with 5 mm diameter subjected to the same treatments as implants. Measurements were performed by a NX10 Park AFM instrument (Park System, Suwon, Korea), equipped with 20-bit closed-loop XY and Z flexure scanners and a non-contact cantilever PPP-NCHR 5M. This instrument implements a True Non-contact™ mode, allowing minimization of the tip-sample interaction, resulting in tip preservation, negligible sample nanotopography modification and reduction of artefacts. On each sample, four different areas were analyzed at a scan rate of 0.1 Hz. Surface topography parameters were obtained using the instrument’s software.

### 4.4. In Vivo Experiment

A total of 18 white male mature New Zealand rabbits, about 2.5 kg of weight, were treated in the investigation. The investigation was approved by the Ethical Committee of the University of Chieti-Pescara, Chieti, Italy, N° 67/2017 del 20-01-2017. A total of 36 implants (4 × 13) were used, 18 sandblasted and double acid etched coated with covalently immobilized collagen type I, (ACTIGEN^®^, Ubigen Srl, Padova, Italy), and 18 uncoated sandblasted and double acid etched implants (Ubigen, Srl, Padova, Italy) were used. A total of eighteen fixtures of each different surface were positioned. The implants were randomly positioned into the articular femoral knee-joint of the animals. Prior to the surgery, the animals were anesthetized with intramuscular infiltrations of fluanizone (0.7 mg/kg b.wt.) and diazepam (1.5 mg/kg b.wt.), and local anesthesia was administered using 1 mL of 2% lidocain/adrenalin solution. In preparation, the skin at the surgical site was washed with soap and water followed by disinfection with an aqueous solution of 10% povidone-iodine (Betadine^®^ Solution, McKesson, Canada). A skin incision with a periosteal flap was elevated to access the articular surface. Dissection of the muscular plane was achieved with blunt scissors, and the knee-joint was exposed using a periosteal elevator. The preparation of the bone implant bed was done with burs under generous saline irrigation. The implant location followed manufacturer’s recommendation, lanceolate drill, 2 mm, and a 3.8 mm under saline irrigation. For inserting the implants, a micromotor was used for each implant. Each rabbit received two implants, one in each knee-joint. Four hours postoperatively the animals were left fasting then food and water ad libidum were offered to the rabbits. During the experimental program, two of the rabbits were substituted.

The animals were euthanized with an intravenous injection of Tanax at 15, 30 and 60 days. A total of thirty-six samples were retrieved. The specimens and surrounding tissues retrieved were stored in 10% buffered formalin and processed to obtain thin ground sections. Then, the samples were dehydrated in a graded series of ethanol rinses and embedded in a glycolmethacrylate resin (Technovit 7200 VLC, Kulzer, Wehrheim, Germany). At the end of the polymerization process, the specimens were sectioned, along the longitudinal axis of the fixture, by a high-precision diamond disc at about 150 µm at slow-speed precision to about 200 µm then ground down to about 40 µm with a specially designed grinding machine Scan 1 Automated System (Pescara, Italy) [20]. A total of three slides were retrieved from each fixture. These slides were stained with toluidine blue and acid fuchsin and observed in normal transmitted light under a Nikon microscope ECLIPSE (Nikon, Tokyo, Japan). New and old bone could be distinguished according to color (light red = old matrix, dark red = new matrix) and values were expressed in percentage (Mean ± SD).

**Figure 12:**
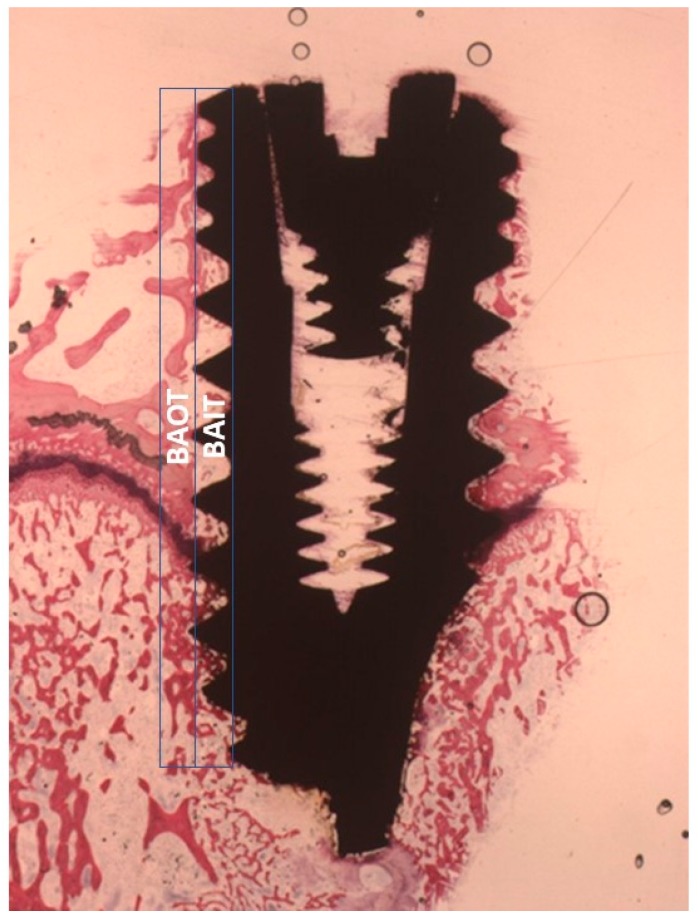
The area directly adjacent to the dental implant was evaluated within the thread part (BAIT), whereas the area far from the implant extended for the same size into the adjacent new/old bone (BAOT).

Bone implant contact (BIC) and bone area inner threads (BAIT) then bone area outer threads (BAOT) were quantified to evaluate the osteogenic parameters around the implant surface and values were expressed in percentage (Mean ± SD). The BAIT in close proximity to the fixture was evaluated within the thread area, while the BAOT, distant to the implant, extended for the same size into the adjacent new/old bone (Figure 12). The different BIC, BAIT and BAOT were measured by a light microscope connected to a high-resolution video camera (16.25-megapixel) (Digital Sight series microscope cameras), a high definition monitor and a personal computer (Notebook Toshiba Satellite pro r50-c-15w). The optical instrument was linked to a dedicated histometry software package able to perform image capturing, recorded by a Sony α330 digital camera and subjected to morphometric analysis by digital image-analysis (NIS-Elements AR 3.0 software, Nikon, Minato, Japan). A total of 18 fixtures for each experimental surface were evaluated in this investigation. 

### 4.5. Micro-CT Analysis

Computed tomography datasets were performed by Skyscan 1172G (Bruker, Kontich, Belgium), a high-resolution 3D imaging system with a L7901-20 Microfocus X-ray Source (Hamamatsu, Japan). 

The acquisition of volumes was performed with 0.5 mm Al filter, image pixel/size of 21.96 µm, camera binning 4 × 4, source voltage of 70 kV, source current of 141 µA, exposure time of 500 ms. The reconstructed tomographic volumes of the acquired scans were acquired by a built-in NRecon Skyscan reconstruction software (Version:1.6.6.0; Skyscan Bruker).

The 3D images were generated using 3D Visualization Softwares CTvox v. 2.5 and DataViewer v. 1.4.4 (Skyscan Bruker) to the volume rendering and virtual sectioning views. The analysis of the samples was carried out using CT-Analyser software version 1.13.

### 4.6. Statistical Evaluation

The statistical evaluation was performed by GraphPad Prism 6 software (GraphPad Software, Inc., San Diego, CA, USA). The outcome data about bone implant contact (BIC), bone area inner threads (BAIT) and bone area outside threads (BAOT) were statistically evaluated between the two experimental groups, and the Student t-Test was applied. The level of significance for the analysis was set at *p* ≤ 0.05. The Pearson correlation test was performed to evaluate the correlation between the bone implant contact (BIC), bone area inner threads (BAIT) and bone area outside threads (BAOT) ratios for each group at 15, 30 and 60 days.

## 5. Conclusions

The outcome of the present research confirms that collagen type I on the implant surface is one of the most efficient approaches for accelerating early osteogenesis and improving the bioactivity of titanium implant surfaces. In the future, bone implant surfaces will be enriched with biomolecules to increase the bone healing process [37,38,39]. In conclusion, these results show that surfaces coated with collagen improve the bioactivity, BIC, and bone around the dental surface compared to control implants, and could be clinically advantageous for shortening the implant healing period.

## Figures and Tables

**Table 1:** Surface composition, as detected by X-ray Photoelectron Spectroscopy (XPS), of Ti and ColTi samples (data are expressed as % at.) and suggests that the thickness of the coating is very thin.

Sample	O	C	N	Ti
Ti	49.2	31.6	0.4	18.8
ColTi	23.8	59.3	14.3	2.7

**Table 2:** Means and SD of the measured Micro-CT BAIT and BOAT ratios and histomorphometric BIC ratios.

		BIC	*p* Value	BAIT	*p* Value	BAOT	*p* Value
15 Days	Uncoated surface	22.4 ± 4.5	*p* = 0.0005	23 ± 0.8	*p* = 0.00004	19 ± 0.8	*p* = 0.00003
	Collagen coated surface	27.5 ± 3.1		31 ± 0.8		21.8 ± 1	
30 Days	Uncoated surface	51.2 ± 3.9	*p* = 0.0021	28 ± 0.8	*p* = 0.00001	36 ± 0.8	*p* = 0.0008
	Collagen coated surface	55.3 ± 3.2		39 ± 2.2		38 ± 2.2	
60 Days	Uncoated surface	56 ± 4.2	*p* = 0.00001	35 ± 2.3	*p* = 0.00006	36 ± 2.3	*p* = 0.0196
	Collagen coated surface	63.6 ± 2.9		42 ± 2.3		44 ± 2.3	

**Table 3:** The Pearson correlation results for the correlation between the Micro-CT BAIT and BAOT and histomorphometric BIC ratios. The Rho (ρ) values is the Pearson rank correlation coefficient. * *p* < 0.05; ** *p* < 0.01

			BIC	BAIT	BOAT
15 Days	Uncoated surface	BIC	-	0.620 *	−0.547 *
		BAIT	0.620 *	-	−0.951 **
		BOAT	−0.547 *	−0.951 **	-
	Collagen coated surface	BIC	-	0.576*	0.578 *
		BAIT	0.576 *	-	0.935 **
		BOAT	0.578 *	0.935**	-
30 Days	Uncoated surface	BIC	-	0.566 *	−0.536 *
		BAIT	0.566 *	-	−0.766 **
		BOAT	−0.536 *	−0.766 **	-
	Collagen coated surface	BIC	-	0.598 *	−0.603 *
		BAIT	0.598 *	-	−0.894 **
		BOAT	−0.603 *	-0.894 **	-
60 Days	Uncoated surface	BIC	-	0.643 *	−0.512 *
		BAIT	0.643 *	-	−0.857 **
		BOAT	−0.582 *	-0.857 **	-
	Collagen coated surface	BIC	-	0.541 *	−0.611 *
		BAIT	0.541 *	-	−0.863 **
		BOAT	−0.611 *	−0.863 **	-
